# Quantitative Ethnobotanical Study of Indigenous Knowledge on Medicinal Plants Used by the Tribal Communities of Gokand Valley, District Buner, Khyber Pakhtunkhwa, Pakistan

**DOI:** 10.3390/plants9081001

**Published:** 2020-08-06

**Authors:** Sulaiman, Sikandar Shah, Sheharyar Khan, Rainer W. Bussmann, Maroof Ali, Dildar Hussain, Wahid Hussain

**Affiliations:** 1 Department of Botany, University of Peshawar, Peshawar 25000, KP, Pakistan; sulaiman097@uop.edu.pk (S.); sikandarbotanist@uop.edu.pk (S.S.); sheharyarbotany@uop.edu.pk (S.K.); 2 Department of Ethnobotany, Institute of Botany, Ilia State University, Tbilisi 0105, Georgia; rainer.bussmann@iliauni.edu.ge; 3 College of Life Science, Anhui Normal University, Wuhu 241000, China; maturi@bs.qau.edu.pk; 4 School of Computational Science Korea Institute for Advanced Study (KIAS), 85 Hoegiro Dongdaemun-gu, Seoul 02455, Korea; 5 Department of Botany, Govt. Post Graduate College, Parachinar 26000, KP, Pakistan

**Keywords:** quantitative study, ethnobotanical, indigenous, conservation, Gokand, Pakistan

## Abstract

The current study on the traditional use of medicinal plants was carried out from February 2018 to March 2020, in Gokand Valley, District Buner, Pakistan. The goal was to collect, interpret, and evaluate data on the application of medicinal plants. Along with comprehensive notes on individual plants species, we calculated Use Value (UV), Relative Frequency of Citation (RFC), Use Report (UR), Fidelity Level (FL), Informant Consensus Factor (FCI), as well as Family Importance Value (FIV). During the current study, a total of 109 species belonging to 64 families were reported to be used in the treatment of various ailments. It included three families (four species) of Pteridophytes, 58 families (99 species) of angiosperm, one family (three species) of Gymnosperms, and two families (three species) of fungi. The article highlights the significance of domestic consumption of plant resources to treat human ailments. The UV varied from 0.2 (*Acorus calamus* L.) to 0.89 (*Acacia modesta* Wall.). The RFC ranged from 0.059 (*Acorus calamus* L. and *Convolvulus arvensis* L.) to 0.285 (*Acacia modesta* Wall.). The species with 100% FL were *Acacia modesta* Wall. and the fungus *Morchella esculenta* Fr., while the FCI was documented from 0 to 0.45 for gastro-intestinal disorders. The conservation ranks of the medicinal plant species revealed that 28 plant species were vulnerable, followed by rare (25 spp.), infrequent (17 spp.), dominant (16 spp.), and 10 species endangered. The traditional use of plants needs conservation strategies and further investigation for better utilization of natural resources.

## 1. Introduction

The inhabitants of remote regions commonly rely upon traditional knowledge concerning medicinal plants for the treatment of many diseases. Plants also provide shelter, food, forage, lumber, and firewood [1]. Moreover, plants also serve to improve air quality, prevent land erosion, and help water recycling. Medicinal plants and plant-based medicines are extensively used in healthcare systems in many developing countries, and also appreciated in many developed countries [2,3,4,5]. Plants provide 80% of the healthcare needs of the world’s population [6,7,8]. The global community has acknowledged that many ethnic communities depend on natural resources, including medicinal plants. The use of plants as traditional therapeutics provides an actual alternative in the healthcare system in evolving nations, especially for rural populations [9,10,11,12]. The investigation of therapeutic plants through qualitative survey methods has become important in recent decades [13,14,15]. The Himalayan region, including parts of Afghanistan, Bangladesh, Bhutan, China, Nepal, Myanmar, India and Pakistan is recognized as a hotspot of biodiversity, for medicinal plant species [16,17,18]. Currently, the USA, China, France, Japan, United Kingdom and Italy are considered to be the largest global markets for medicinal plants. Although most countries in Asia harvest medicinal plants for their internal traditional uses, very few, especially China, India, Indonesia, Nepal, Bangladesh, Iran and Pakistan, are capable of producing them in commercial quantity. Pakistan ranks as the 7th producer of medicinal plants in Asia [18,19]. In Pakistan, around 600 species are used as traditional medicine, and more than 75% of the local population relies upon therapeutic herbs for all or the majority of their healthcare needs. The medicinal flora is extensively utilized in the manufacturing of medicines, food, cosmetics, and dietary supplements [20]. Most of the indigenous populations still depend on plant-derived medicines [21,22]. Herbal treatments have an ancient of utilization in East Asia [23], and are believed to have few side effects and high efficiency [24,25].

*Viola pilosa* Blume, *Diospyros lotus* L., *Morchella esculenta* Fr., *Trillium govanianum* Wall. Ex D. Don are the most important medicinal species produced in Pakistan [26]. The knowledge of medicinal species has mostly been transmitted orally from generation to generation [27]. Cultural practices and local biodiversity are the driving factors of medicinal species are utilization [28,29]. Ethnomedicinal research can serve as the basis for the development of new natural remedies for native plant species [30,31,32,33,34]. In Pakistan, local market systems named “Pansar” specifically deal with medicinal species, including the export of important quantities of plants [16,35]. The utilization of plants as medication varies from 4 to 20% in various nations, and around 2500 species are traded globally [25]. In Pakistan alone, about 50,000 Ayurvedic specialists, tabibs (experts of Unani medicine), and many unregistered health experts are working in far-flung mountainous and urban areas making common use of around 200 plant species in herbal medications [36,37]. Botanical gardens, universities, and the National Council for Tibb, Hamdard, and Qarshi industries help to control and develop the herbal industry in Pakistan. There are over 4000 registered manufacturers of herbal marketable products in the country [38,39,40].

The present study addresses the issue of ethnobotanically important plants as an important source for the treatment of many ailments in the Gokand Valley. Extensive surveys, interviews and interactions with local healers focused on the reliability, efficacy and spectrum of medicinal plant use among local residents, and the economic impact of such use. In our approach, we hypothesized that: (1) Plant use, especially for medicinal purposes, was still highly important in this remote area; (2) local knowledge, although part of a common cultural sphere, would differ from neighboring areas; and (3) pressure on the resources was increasing.

## 2. Materials and Methods

### 2.1. Study Area and Climate

**Figure 1:**
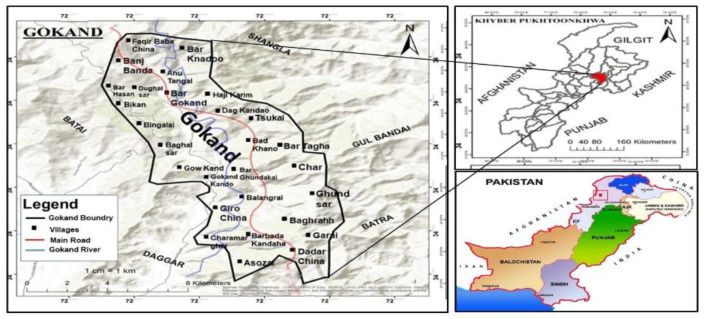
Map of the research area.

Gokand Valley, District Buner is located in the North of Province Khyber Pakhtunkhwa. It lies between 34°09′ 34°43′ N and 72°10′ 72°47′ E, covering an area of 1760 km^2^ (Figure 1). The region is surrounded by Swat and Shangla to the North, by Malakand Agency to the west, by Mardan District to the south and by Hazara Division to the east [41]. The climate of the lower regions of the valley is sub-tropical, and the upper regions are temperate. The summer is moderately hot and short while the winter season is cool and extends from October to March [42]. Due to high rainfall people migrate from the upper to the lower regions till the fall in average temperature. River Barandu the largest important water channel passes by the majority of the villages in the area. The maximum rainfall recorded during February and March is 289.1 mm, while 540.6 mm of precipitation falls in the rest of the year [43].

### 2.2. Data Collection

Ethnomedicinal information was collected from February 2018 to March 2020. A purposive sampling method was used for the selection of informants, with local participants pointing out people they thought had specific medicinal plant knowledge, and information regarding the ethnomedicinal use of plants was collected through semi-structured open-ended interviews with a standard questionnaire. The interviews were carried out face-to-face with individual participants, and also in groups discussions. The respondents were briefed about the aims and objectives of the study, and the prior informed consent of each participant was obtained. The work followed the International Society of Ethnobiology Code of Ethics (International Society of Ethnobiology. International Society of Ethnobiology Code of Ethics (with 2008 additions). 2006; http://www.ethnobiology.net/what-we-do/core-programs/ise-ethics-program/code-of-ethics/code-in-english/). All the interviews were conducted in Pashto, the local language of the communities. The final selection of respondents was primarily based on their knowledge and willingness to share information. The questionnaire included data on; demographic features of the informants, vernacular names of plants, parts used, availability, route of application of plants and diseases treated.

### 2.3. Demographic Data of Local Participants

In the present study a total of 168 respondents, including 37 farmers, 33 homemakers, and non-professional elders, 29 plants gatherers, 26 shepherds, 14 healers, 15 hunters, 9 dealers, and 5 salespersons, were interviewed using open-ended questionnaires, face to face interviews and group discussions. Respondents of different professions and various age groups were interviewed in various seasons of the year. The age of the informants ranged from less than 20 years to above 60 years. Thirty-three informants were between 21–40 years old, while 62 informants were above 60 years old. Among the four groups of male informants, 11 were less than 20, 22 aged 21–40, 38 between 41–60, and 50 aged above 60 years. Of the female respondents, four were in the age group below 20, 11 in the age group 21–40, 20 in the age group 41–60, and 12 were over 60 years old. The majority of the local population belonged to rural areas (78.57%) and depended mainly on agricultural production (Table 1).

### 2.4. Plant Collection and Identification

Plants species cited for a specific disease in the area were collected, pressed and mounted on herbarium sheets for correct identification. The specimens were identified by taxonomists at the Department of Botany, University of Peshawar, and with the help of the Flora of Pakistan [44,45,46], and deposited in the Herbarium Department of Botany, University of Peshawar.

### 2.5. Statistical Data Analysis

The data collected were analyzed statistically using various quantitative indices: Use Value (UV), Relative Frequency of Citation (RFC), Use Report (UR), Fidelity Level (FL), Informants Consensus Factor (ICF), and Family Importance Value (FIV).

#### 2.5.1. Use Value

Use value is used to determine the relative importance of plant species. It is calculated using the use-value formula:

(1) $$UV=\frac{\sum_{1}^{n}U_{i}}{N},\;$$

where ‘*UV_i_*’ is the frequency of citations for species through all respondents and ‘Ni’ the number of respondents [47,48].

#### 2.5.2. Relative Frequency of Citation and Use Reports

Relative Frequency Citation (*RFC*) was used to record the highest therapeutic medicinal flora of the valley, which is consumed for the treatment of numerous ailments.

(2) $$RFC=\frac{FC}{N}\;\left(0<RFC<1\right).$$

*RFC* shows the importance of each species and is given by the frequency of citation *FC*, the number of respondents (*N*) in the survey as used by [48,49].

#### 2.5.3. Fidelity Level

The Fidelity Level is the percentage of respondents who cited the uses of specific plant species to treat a specific disease in the research area. The FL index is calculated as;

(3) $$FL\left(\%\right)=\frac{N_{p}}{N}\times 100$$

where “*N_p_*” is the specific Number of citations for a particular ailment, and ‘*N*’ is the total number of informants mentioned the species for any disease [50].

#### 2.5.4. Informant Consensus Factor

The Factor Consensus Informants (*FCI*) was used to evaluate the consent of respondents about the use of plant species for the treatment of various ailments categories.

(4) $$FCI=\frac{N_{t}}{N_{ur}}.$$

where *N_ur_* = number of use reports from informers for a disease category treated by a plant species; *N_t_* = number of species or taxa used for treating that disease category. FIC value ranges from 0 to 1. Where 1 represents the highest value of respondents, and 0 indicates the lowest value [51].

#### 2.5.5. Family Importance Value

Family Importance Value (*FIV*) was used to determine the relative importance of families. It was calculated by taking the percentage of informants mentioning the family.

(5) $$FIV=\frac{FC}{N}\times 100,$$

where *FC* is the number of informers revealing the family, while *N* is the total number of informants participating in the research [48].

### 2.6. Conservation Status

The Conservation status was reported for species growing wild in the area. The information was recorded and collected for different conservations attributes by following International Union for Conservation and Nature [52]. Plants were classified using International Union for Conservation of nature (IUCN) Criteria, 2001 as displayed in Table 2.

## 3. Results

### 3.1. Medicinal Plant Taxonomy and Growth Forms

During the current study, a total of 109 species belonging to 64 families were reported to be used in the treatment of various ailments. It included three families (four species) of Pteridophytes, 58 families (99 species) of angiosperm, one family (three species) of Gymnosperms, and two families (three species) of fungi. The species reported along with their botanical name, local name, voucher number, family, part used, preparation of remedies, route of administration, medicinal uses, frequency of citation, and relative frequency of citation with their conservation status are presented in (Table 3). The most dominant families in the term of the maximum of reported taxa were Asteraceae (six species), followed by Lamiaceae, Moraceae, and Rosaceae with five species each. The literature confirmed that Asteraceae, Lamiaceae, Moraceae, and Rosaceae were the most widely recognized therapeutic families. The most often-cited taxa were *Acacia modesta, Oxalis corniculata, Mentha longifolia, Morchella esculenta, Withania somnifera*, and *Zanthoxylum armatum*, due to their efficiency, accuracy, and easy availability.

Herbs were the most commonly used life form, with 57 reports (52.29%), followed by shrubs which had 27 reports (24.77%) and trees with 25 reports (22.93%). The common use of wild herbs may be due to their easy accessibility and efficacy in the treatment of various diseases, compared to other life forms.

### 3.2. Plant Parts Used and Preparation of Remedies

**Figure 2:**
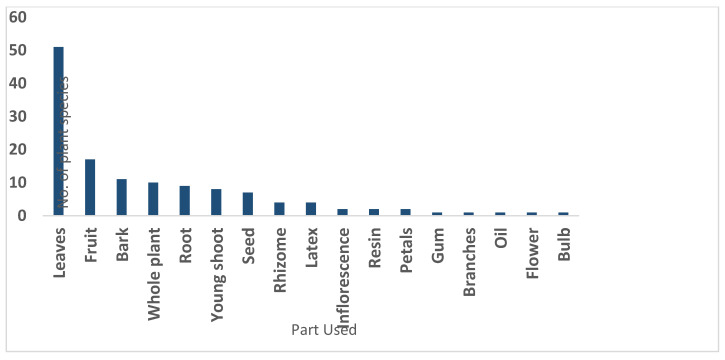
Plant parts used in the preparation of remedies.

Based on a total of 3297 use reports, the part of the plants most frequently used for treating different diseases were leaves (38.63%), followed by fruit (12.87%), bark (8.33%) and whole plants (7.57%), as shown in Figure 2. The least reported plant part used was gum, bulb, oil, branches and flower, each with 1 percent.

**Figure 3:**
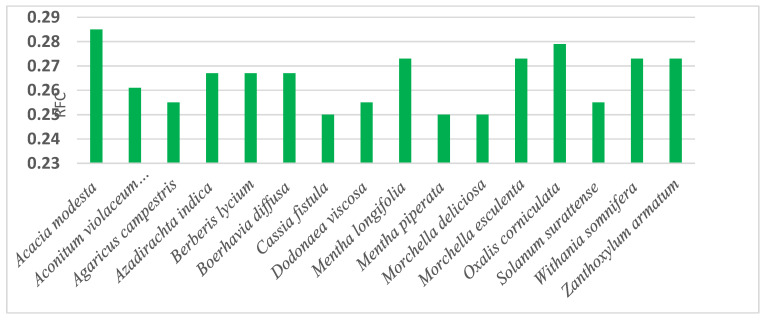
Medicinal plants and fungi with Highest Relative Frequency Citation.

The most commonly used method of preparation was decoction (29%), followed by juice (21%), direct and poultice, each with 16% (Figure 3). The most common method of administration of herbal recipes is decoction in different parts of the world.

### 3.3. Availability and Mode of Administration

During the collection of data, most participants said that therapeutic flora were commonly collected from various kinds of habitats, such as forests, deserts, and hilly areas. The most common route of administration of remedies was oral (66%), followed by both oral and topical (20%), topical (11%) and 1% each as toothbrush, inhaled and eardrop.

### 3.4. Quantitative Ethnomedicinal Data Analysis

The present work was the first ever study to record quantitative data of the medicinal flora of the region, including Relative Frequency of Citation, Use Value, Use Report, Fidelity Level, Informant Consensus Factor and Family Importance Value.

#### 3.4.1. Use Value

The UV of the encountered plant species ranged from 0.2 to 0.89. The highest UV was found for *Acacia modesta*, while lowest was for *Acorus calamus.* Other important plant species with high use value were *Morchella esculenta* (0.86), *Oxalis corniculata* (0.85), *Zanthoxylum armatum* (0.84), *Mentha longifolia* (0.82), *Withania somnifera* (0.80), *Azadirachta indica* (0.80), *Berberis lycium* (0.77), *Aconitum violaceum* (0.68), *Agaricus campestris* (0.65), *Adiantum capillus-veneris* (0.65), *Dodonaea viscosa* (0.60) *and Solanum surattense* (0.58). It was also observed that the highest use values were due to the high number of use reports in the study area.

#### 3.4.2. Relative Frequency of Citation and Use Report

The RFC value ranged from 0.059% to 0.285% for healing uses of the medicinal plants. The majority of the respondents reported a total of 16 plant species for different treatment purposes. The highest value of RFC was recorded for *Acacia modesta* (0.285%), followed by *Oxalis corniculata* (0.279%), *Mentha longifolia, Morchella esculenta, Withania somnifera* and *Zanthoxylum armatum* (0.273%) each. The gum of *Acacia modesta* was used for the treatment of hepatitis and as muscle relaxant. The next highest RFC was calculated for *Oxalis corniculata* with medical indications, such as stomach disorders, skin inflammation, and for removal of warts. Among the remaining four plants, *Mentha longifolia* is used for diarrhea, vomiting, and abdominal pain; *Morchella esculenta* for infertility and as a tonic; *Withania somnifera* for Pneumonia and as diuretic; and *Zanthoxylum armatum* for gum pain, abdominal pain and as cooling agent. The lowest RFC value was 0.059%, recorded for *Acorus calamus* and *Convolvulus arvensis*.

In the present study, Use report value varied from 2 to 43. Acacia modesta, Mentha longifolia, Oxalis corniculata, Morchella esculenta, Withania somnifera, Zanthoxylum armatum, Azadirachta indica, Berberis lycium, Aconitum violaceum, Agaricus campestris, Dodonaea viscosa and Solanum surattense were the most used plant species.

#### 3.4.3. Fidelity Level

Fidelity level highlights the medicinal flora, Medicinal plants with maximum curative properties have the highest fidelity level, i.e., 100%. In the present investigation, the FL varied from 20 to 100%. The plant species most commonly utilized in the research area with 100% fidelity levels were *Acacia modesta* and *Morchella esculenta*, which were used to treat hepatitis and infertility, respectively. The FL determined for, *Oxalis corniculata* (stomach disorders), *Mentha longifolia* (Diarrhea), *Zanthoxylum armatum* (abdominal pain), *Azadirachta indica* (Hepatitis), *Withania somnifera* (Pneumonia), *Berberis lycium14* (Diabetes), and *Aconitum violaceum* (Arthritis) were 97.87, 95.65, 95.65, 95.55, 93.47, 93.33 and 86.36% respectively.

#### 3.4.4. Informant Consensus Factor

The inhabitants used medicinal plants in the treatment of 64 health disorders. The important disorders were hepatitis, diabetes, diarrhea, dysentery, hypertension, anemia, arthritis, infertility and ulcer. To determine the informant consensus factor (FCI), all the reported ailments were first grouped into 15 different disease categories on the basis of their use reports (Table 4). The uppermost FCI value is reported for gastro-intestinal disorders (0.45), followed by respiratory disorders, glandular disorders (0.44) and cardiovascular disorders (0.43). Amongst the four major disease categories, gastro-intestinal disorders dominated with 137 use-reports, followed by respiratory disorders, glandular disorders, and cardiovascular disorders (110, 104, and 82 use reports, respectively) as mentioned in Table 4. About 68.8% plants were used to treat gastro-intestinal disorders, followed by respiratory disorders (56.88%), glandular disorders (53.21%), cardiovascular disorders (43.11%) analgesic, antipyretic, and refrigerant (32.11%), Dermatological disorders (29.35%), hepatic disorders (24.77%), body energizers (23.85%), and urologic disorders (20.18%). These results show that gastro-intestinal and respiratory disorders were especially common in the study area.

#### 3.4.5. Family Importance Value (FIV)

**Figure 4:**
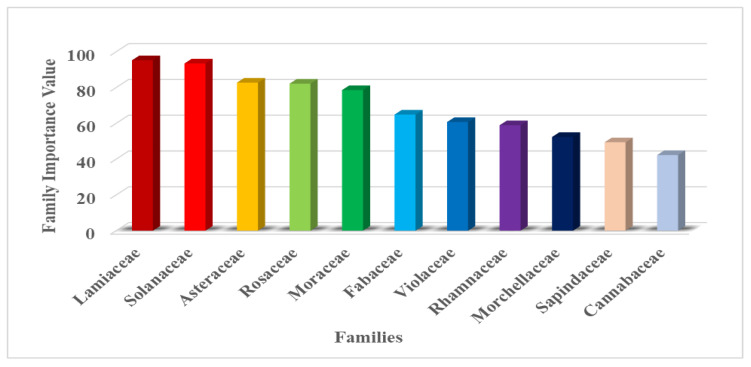
Family Importance Values.

The importance of a plant family increases with the increase in the frequency of citations of all species. Figure 4 represents 11 plant families with maximum FIV, amongst which Lamiaceae was the leading family (95.23%), followed by Solanaceae (93.45%), Asteraceae (82.73%), and Rosaceae (82.14%). Acoraceae has the lowest family importance value, with 5.95% (Table 5).

### 3.5. Conservation Status of the Medicinal Flora

**Figure 5:**
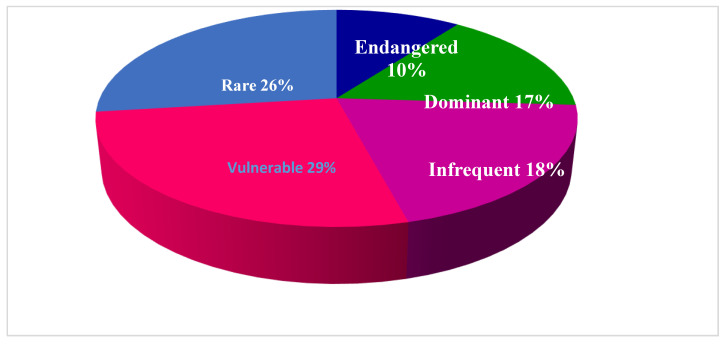
Conservation status of medicinal plants.

In recent times, global conservation of threatened plant diversity has gradually increased, and governments around the world have been working on this issue. Climate change, human-caused habitat change, and the introduction of exotic plants are considered among the main drivers for habitat loss and species extinction. Therefore, ex-situ conservation is recommended for endangered species. The same holds true for the study area, but until now no project has been started for the conservation of forests or vegetation. Consistent with IUCN Red List Criteria (2001) the conservation status of the 96 wild medicinal species encountered was assessed, and 28 species were found to be vulnerable followed by 25 that were rare, 17 infrequent and 16 dominant, respectively, as shown in Figure 5. We found that 10 species were endangered in the study area, due to urbanization, small size population, anthropogenic activities, much collection, marble mining and adverse climatic conditions. The remaining 11 plants were cultivated, and 1 plant (*Broussonetia papyrifera)* was invasive. The lack of suitable habitat and unsustainable use have already affected their regeneration and put them into the endangered category. Indigenous knowledge can also contribute to sustainable use and conservation of important medicinal plant species.

## 4. Discussion

Medicinal plant research in Asia continues to receive significant national and international attention, particularly with regard to its multiple roles in poverty alleviation and health care support. Nine countries (China, Korea, India, Indonesia, Malaysia, Myanmar, Sri Lanka, Thailand, and Vietnam) have already published their National Monographs for herbal drugs, while official herbal pharmacopeias exist in Bangladesh, India, Indonesia, Sri Lanka, Thailand, and Vietnam. In general, there is increased interest by practitioners to implement medicinal plant management and usage practices. Traditional treatments are often a gender-based occupation which both men and women perform [53]. Medicinal knowledge is still mostly passed on from one generation to another with time [54]. This suggests an urgent need for scientific investigations of these process. It is clear that comprehensive information on (formal and informal) is important for establishing sound guidelines for medicinal plants production, use, commercialization and management [18,19]. In the surrounding areas of Gokand Valley, i.e., Malakand and Swat, medicinal plants have already been documented in detail [55,56], and like in our study area, medicinal plant species were extensively used. In such earlier studies, leaves were also favored in traditional approaches [36,57,58]. In general, herbal medicines were prepared from a single plant species [10,14]. However, in some cases, more than one plant species was used in traditional recipes [59]. Around 80% of the people in emerging economies depend on therapeutic plants to treat ailments [60,61]. Indigenous healers are vital to meet the basic health requirements of local populations, not only in the study area. The medicinal plants with maximum UV required protection for sustaining biodiversity in the investigation region. However, no program or project for the maintenance and conservation of flora and vegetation is functioning in the study area, urbanization, marble mining, overharvesting and grazing were detected as the main threats to therapeutic plant species.

### 4.1. Ethnopharmacological Relevance

People have long histories in the uses of traditional medicinal and aromatic plants for medical purposes in the world, and currently, this use is often actively promoted. The medicinal significance of these plants can be authenticated through ethnomedicinal research, and a variety of studies have confirmed the use of medicinal species. *Justicia adhatoda* leaf extract is used for injuries and in joint pain [25]*. Artemisia vulgaris* is used against intestinal worms and for cardiac problems [25]. Leaves, roots and bark of *Berberis lycium* are used for diabetes, muscle growth, broken bones, and diarrhea [19,62]. Traditionally, *Aconitum violaceum* helps to remedy cough, asthma, neural disorders and heart disorders, as well as for treatment of joint pain and sciatica [63]. *Cichorium intybus* is used against gastro-intestinal problems, asthma, and gall stones [64]. Ethnobotanical research in neighboring countries also supported our research findings. *Tribulus terrestris* is used as a blood purifier, *Nerium oleander* for toothache, and scorpion stings [8,65]. The above ethnomedicinal information and similarities with other regions confirm the importance of the described plants. *Broussonetia papyrifera* has long been used for the treatment of inflammation in Chinese medicine, particularly to treat respiratory inflammation [66]. The extract of *Mentha longifolia* is used against infertility, Dyspepsia and Diarrhea because of the existence of Alkaloids, Tannins and Flavonoids [67]. The ethanolic extract of *Mentha piperata* is used to treat nausea, indigestion and anorexia [68]*. Tribulus terrestris* extracts are commercially marketed and use for the development of muscles, sore throat, mental stimulation, relaxing the period of uncontrolled climacteric in women, and digestion disorders [69]. *Withania somnifera* contains Withanolide A and Withaferin A and appears to possess various therapeutic activities against diseases like Alzheimer’s, cancer, fluid retention, Parkinson’s disease and diabetes [70]. *Cannabis sativa* contains Cannabidiol, which is used as an antipsychotic, schizophrenic agent and anxiolytic [71]. The pure leaf extract of *Olea ferruginea* has special inhibitory effects on fungal and bacterial pathogens [72]. Ethanolic leaves extract of *Acacia modesta* showed significant activity against *E. coli*, *Proteus mirabilis, S. aureus, K. pneumonia, P. aeruginosa, S. typhi, B. cereus and B. subtilis and Streptococcus pneumonia* [73]. This shows that further research on the reported ethnomedicinal plant species can lead to the recognition of novel agents with useful properties.

### 4.2. Novelty and Future Impacts

The comparison of our study with the ethnomedicinal literature indicated that neighboring areas [41,42,43], while the more distant areas had comparatively fewer similarities, due to cultural and traditional differences—thus, confirming our respective hypothesis. The comparative study between previously reported medicinal plants showed that six plant species, *Aconitum violaceum, Broussonetia papyrifera, Cedrus deodara, Celtis caucasica, Conandrium arnotaimum,* and *Pinus wallichiana*, were not previously documented in this area for their medicinal values. The newly reported plants (and their uses) were *Aconitum violaceum* (arthritis), *Broussonetia papyrifera* (diarrhea), *Cedrus deodara* (cooling and antipyretic), *Celtis caucasica* (wound healing), *Conandrium arnotaimum* (skin allergy), and *Pinus wallichiana* (antipyretic). These plant species might provide leads for pharmacological activities and detection of bioactive compounds in search of new drugs. The study also highlighted nine species of antipyretic plants, such as *Ajuga bracteosa*, *Cedrus deodara, Mentha spicata, Pinus roxburghii, Pinus wallichiana, Rhododendron arboretum, Solanum surattense, Sonchus asper*, and *Tinospora cordifolia* and nine plant species to treat arthritis, such as *Achyranthes aspera, Aconitum violaceum,* *Asparagus racemosus*, *Buxus wallichiana, Cuscuta reflexa, Hedera helix,* *Justicia adhatoda, Rumex hastatus*, and *Viola canescens.* Such a large number of plant species for antipyretics and arthritis pain had not previously reported anywhere in Pakistan. Sadly, many ethnobotanical studies reveal either a dramatic or gradual loss of traditional knowledge and practices [72].

## 5. Conclusions

The current study showed that the area has a substantial diversity of medicinal plants; utilization of medicinal plants and plant-based remedies is abundant in the area. Total of 109 medicinal species, from 64 families were documented for the treatment of 64 various ailments. *Aconitum violaceum, Broussonetia papyrifera, Cedrus deodara, Celtis caucasica, Conandrium arnotaimum,* and *Pinus wallichiana*, were reported for the first time from the study area for the treatment of arthritis, diarrhea, torridity (cooling agent), wound healing, skin allergy, and as antipyretic, respectively. This confirmed our first hypothesis—that plants used especially for medicinal purposes are still highly important in this remote area. The people of the study area are economically very deprived, and their main occupation is agriculture, work as laborers, home-run shops and engaged in livestock rearing. The terrain of Gokand valley is hilly, and most of the villages of the region are cut off from frequent visits to town and inhabitants of the area still depend on the medicinal plants for the basic health requirements. In the surrounding areas of Gokand Valley, i.e., Malakand and Swat, medicinal plants have already been documented in detail; but in current study, medicinal plant species were reported with different uses. This confirmed our second hypothesis, that the local knowledge in the research area would show distinct differences to surrounding areas. Around 80% of the people in emerging economies depend on therapeutic plants to treat ailments. Indigenous healers are vital to meet the basic health requirements of local populations. The medicinal plants with maximum UV required protection for sustaining biodiversity in the investigation region. Anthropogenic activities, such as urbanization, marble mining, overharvesting, and grazing, were detected as the main threats to local biodiversity, and this, together with increasing market demand, puts increased pressure on plant resources, as we assumed in our third hypothesis. The projects of cultivation of medicinal plants must be implemented to eliminate their extinction in the area under study.

## Figures and Tables

**Table 1:** Demographic details.

Variables	Categories	Number of Informants	Percentage	Sum of Reports
Gender ratio	Men	47	27.976	588
	Women	121	72.023	2709
Age	<20	15	8.928	107
	21–40	33	19.642	217
	41–60	58	34.523	409
	>60	62	36.904	2564
Educational Background	Illiterate	67	39.88	1682
	Matric	53	31.547	838
	Intermediate	26	15.476	352
	Graduate	17	10.119	300
	Postgraduate	5	2.976	125
Informant category	Farmer	37	22.023	1455
	Elder (housewives and non-professional)	33	19.642	930
Profession	Shepherd	26	15.476	130
	Plant gatherer	29	17.261	195
	Healer	14	8.333	453
	Hunter	15	8.928	85
	Salesperson	5	2.976	11
	Dealer	9	5.357	38
Life type	Urban area	36	21.428	-
	Hilly area	132	78.571	-

**Table 2:** IUCN Criteria, 2001 for conservation classes.

Availability	Collection
0 = Uncommon or very rare	0 = More than 1000 kg/year
1 = Less common or rare	1 = Consumed from 500–1000 kg/year
2 = Occasional	2 = Consumed from 300–500 kg/year
3 = Abundant	3 = Consumed from 100–200 kg/year
**Growth**	**Part used**
0 = Regrowth in more 3 years	0 = Root/Whole
1 = Regrowth within 3 years	1 = Bark
2 = Regrowth within 2 years	2 = Seeds, Fruits
3 = Regrowth within 1 year	3 = Flowers
4 = Regrowth in a season	4 = Leaves/Gum/Latex
**Total Score**	
0–4	Endangered
5–8	Vulnerable
9–12	Rare
13–14	Infrequent
15–16	Dominant

**Table 3:** Medicinal plant species and fungi in Gokand Valley, Buner, Khyber Pakhtunkhwa, Pakistan.

Family	Botanical Name Local Name Voucher Number	Availability	Habit	Part(s) Used	Preparation of Remedies	ROA	Medicinal Uses	FC	RFC	UR	UV	FL	CS
**Pteridophytes**													
Dryopteridaceae	Dryopteris juxtaposita Christ. Kwangai B.Sul.059.UOP	W	H	Young shoot	Juice	O	Bone weakness, dyspepsia	43	0.255	22	0.51	65.11	E
Equisetaceae	*Equisetum arvense* L. Bandaky B.Sul.061.UOP	W	H	Whole plant	Juice	O	Kidney stones	26	0.154	11	0.42	57.69	V
Pteridaceae	*Adiantum capillus-veneris* L. Sumbal B.Sul.025.UO	W	H	Whole plant	Decoction	O	Constipation, pneumonia, scorpion bite	23	0.136	15	0.65	56.52	E
	*Adiantum venustum* D. Don Parsohan B.Sul.026.UOP	W	H	Whole plant	Decoction	O	Scorpion bite, constipation	32	0.19	18	0.56	31.25	V
**Angiosperm**													
Acanthaceae	*Justicia adhatoda* L. Bekar B.Sul.071.UOP	W	S	Leaves	Decoction, poultice	O, top.	Wound, swelling, arthritis, headache	30	0.178	13	0.43	50	D
Acoraceae	*Acorus calamus* L. Skha waga B.Sul.024.UOP	W	H	Rhizome	Decoction	O	Menstrual cycle regularity, dyspepsia	10	0.059	2	0.2	20	R
Amaranthaceae	*Achyranthes aspera* L. Ghishkay B.Sul.022.UOP	W	H	Leaves	Juice	O	Stomachache, arthritis, diarrhea	15	0.089	4	0.26	46.66	I
	*Amaranthus viridis* L. Chalwery B.Sul.032.UOP	W	H	Leaves	Juice, poultice, vegetable	O, top.	Urinary diseases, hair tonic	12	0.071	5	0.41	33.33	D
Amaryllidaceae	*Allium jacquemontii* Kunth Oraky B.Sul.031.UOP	W	H	Leaves	Roast, decoction	O	Blood pressure, abdominal pain	24	0.142	12	0.5	62.5	R
	*Narcissus poeticus* L. Gul-nargus B.Sul.082.UOP	W	H	Bulb	Juice	O	Allergy, pimples	33	0.196	13	0.39	54.54	C
Anacardiaceae	*Pistacia integerrima* J.L. Stewart ex Brandis Kharawa B.Sul.094.UOP	W	T	Bark	Decoction	O	Hepatitis, loss of appetite	43	0.255	22	0.51	65.11	R
Apiaceae	*Pimpinella diversifolia* DC. Tarpakai B.Sul.091.UOP	W	H	Leaves	Decoction	O	Fever, stomachache, emphysema	28	0.166	13	0.46	50	V
Apocynaceae	*Caralluma edulis* (Edgew.) Benth. Ex Hook.f. Famanhy B.Sul.046.UOP	W	H	Whole plant	Decoction, powder	O	Anti-peristalsis, otitis media	37	0.22	14	0.37	51.35	R
	*Nerium oleander* L. Ganderay B.Sul.084.UOP	C	S	Leaves	Roast	O	Anti-microbial, tooth ache	21	0.125	10	0.47	66.66	---
Araliaceae	*Hedera helix* L. Payo zela B.Sul.068.UOP	W	S	Leaves	Decoction	O	Diabetes, arthritis	35	0.208	18	0.51	51.42	V
Asclepiadaceae	*Calotropis procera* (Aiton) Dryand. Spalamy B.Sul.043.UOP	W	S	Leaves	Powder	O	Digestion, flatulence	21	0.125	11	0.52	66.66	E
Asparagaceae	*Asparagus officinalis* L. Tendory B.Sul.035.UOP	W	S	Root, young shoot	Juice	O	Fever, flatulence, kidney stones	23	0.136	10	0.43	47.82	V
	*Asparagus racemosus* Willd. Gangra boty B.Sul.036.UOP	W	H	Leaves	Decoction	O, top.	Arthritis, skin diseases	19	0.113	7	0.36	42.10	V
Asphodelaceae	*Asphodelus tenuifolius* Cav. Ogaky B.Sul.037.UOP	W	H	Leaves	Decoction	O	Blood pressure, tension	38	0.226	17	0.44	55.26	R
Asteraceae	*Artemisia vulgaris* L. Tarkha B.Sul.034.UOP	W	S	Root, leaves	Poultice	top.	Skin diseases, Intestinal worms	38	0.226	18	0.47	47.36	E
	*Cichorium intybus* L. Harn B.Sul.051.UOP	W	S	Leaves	Decoction	O	Asthma, indigestion	32	0.19	13	0.40	56.25	V
	*Senecio chrysanthemoides* DC. Sra jaby B.Sul.107.UOP	W	H	Leaves, rhizome	Poultice	top.	Swelling, wound healing	27	0.16	13	0.48	62.96	I
	*Sonchus asper* L. Hill Shodapy B.Sul.110.UOP	W	H	Leaves	Decoction	O	Stomach problems, antipyretic	15	0.089	5	0.33	40	R
	*Taraxacum officinale* F.H. Wigg. Ziar guly B.Sul.114.UOP	W	H	Leaves, petals	Decoction	O	Cough, yellowness of skin eyes and urine	18	0.107	8	0.44	38.88	D
	*Xanthium strumarium* L. Ghishky B.Sul.126.UOP	W	S	Leaves, seeds	Decoction, Poultice	O, top.	Indigestion, diarrhea, smallpox	9	0.053	3	0.33	22.22	D
Berberidaceae	*Berberis lycium* Royle Kwary B.Sul.039.UOP	W	S	Leaves, root, bark	Poultice, decoction	O, top.	Diabetic, wound healing, bone fracture, pain, diarrhea	45	0.267	35	0.77	93.33	V
Boraginaceae	*Trichodesma indicum* (L.) Lehm. Ghwa jaby B.Sul.118.UOP	W	H	Root	Poultice	top.	Anti-inflammatory, snake bite	38	0.226	17	0.44	50	R
Brassicaceae	*Capsella bursa-pastoris* (L.) Medik. Bambysa B.Sul.045.UOP	W	H	Leaves, root	Juice	O	Tension, anxiolytic	33	0.196	12	0.36	45.45	V
	*Nasturtium officinale* R.Br. Talmera B.Sul.083.UOP	W	H	Root, young shoot	Juice, vegetable	O	Wound healing, toothache	26	0.154	12	0.46	46.15	D
Buxaceae	Buxus wallichiana L. Shamshad B.Sul.042.UOP	W	S	Leaves	Powder, decoction	O, top.	Arthritis, bone fracture, purgative	39	0.232	19	0.48	51.28	V
Cactaceae	*Opuntia dillenii* (Ker Gawl.) Haw. Tohar B.Sul.086.UOP	W	H	Fruit	Juice	O	Anemia	40	0.238	21	0.52	70	D
Caesalpiniaceae	*Cassiafistula* L. Amaltas B.Sul.047.UOP	W	T	Fruit	Decoction	O	Constipation, skin infection, fever	42	0.25	22	0.52	76.19	R
Cannabaceae	*Cannabis sativa* L. Bang B.Sul.044.UOP	W	H	Leaves	Smoke, Poultice	Inhale	Sedative, narcotic, ulcer, pain killer	34	0.202	16	0.47	70.58	D
	Celtis caucasica L. Tagha B.Sul.049.UOP	W	T	Bark, fruit	Decoction, Poultice	O, top.	Wound healing, burning	37	0.22	17	0.45	54.05	V
Caryophyllaceae	*Stellaria media* L. Vill. Spin goly B.Sul.112.UOP	W	H	Leaves	Poultice	top.	Bone fracture	32	0.19	10	0.31	40.62	I
Celastraceae	*Gymnosporia royleana* Wall. Sor azghay B.Sul.067.UOP	W	S	Fruit	Direct	O	Blood purifier, gum pain	28	0.166	10	0.35	39.28	D
Chenopodiaceae	*Chenopodium album* L. Srmai B.Sul.050.UOP	W	H	Young shoot	Decoction	O	Hepatitis, constipation	19	0.113	10	0.52	52.63	R
Convolvulaceae	*Convolvulus arvensis* L. Prewatai B.Sul.053.UOP	W	H	Leaves	Powder	O	Pimple, acne, stomach problems	10	0.059	4	0.4	30	I
	*Cuscuta reflexa* Roxb. Nary zaila B.Sul.054.UOP	W	H	Young shoot	Juice	O	Arthritis, blood purifier	13	0.077	6	0.46	38.46	I
Dryopteridaceae	Dryopteris juxtaposita Christ. Kwangai B.Sul.059.UOP	W	H	Young shoot	Juice	O	Bone weakness, dyspepsia	43	0.255	22	0.51	65.11	E
Equisetaceae	*Equisetum arvense* L. Bandaky B.Sul.061.UOP	W	H	Whole plant	Juice	O	Kidney stones	26	0.154	11	0.42	57.69	V
Ericaceae	*Conandrium arnotaimum* L. Sra makha B.Sul.052.UOP	W	S	Whole plant	Decoction	O	Skin allergy	30	0.178	15	0.5	60	E
	*Rhododendron arboreum* Sm. Gul-namaire B.Sul.099.UOP	W	S	Flower	Juice	O	Antipyretic	27	0.16	14	0.51	59.25	I
Euphorbiaceae	*Euphorbia helioscopia* L. Mandanro B.Sul.062.UOP	W	H	Latex	Powder	O	Kidney stone, cholera	23	0.136	12	0.52	69.56	D
	*Euphorbia hirta* L. Wrmago B.Sul.063.UOP	W	H	Latex	Poultice, juice	O, top.	Kidney stone, bronchitis, constipation	30	0.178	15	0.5	60	I
	*Mallotus philippensis* (Lam.) Müll.-Arg. Kambela B.Sul.072.UOP	W	T	Bark, seed	Juice, direct	O	Stomach pain	16	0.095	8	0.5	56.25	R
Fabaceae	*Acacia modesta* Wall. Palosa B.Sul.021.UOP	W	T	Gum	Direct	O	Relaxant, hepatitis	48	0.285	43	0.89	100	R
Fabaceae	*Indigofera gerardiana* Wall Ghoreja B.Sul.069.UOP	W	S	Root	Direct	O	Stomach ache	38	0.226	16	0.42	47.36	V
Fabaceae	*Robinia pseudoacacia* L. Kikar B.Sul.100.UOP	C	T	Leaves, Inflorescence	Decoction, poultice	O, top.	Spasm, diabetes	23	0.136	9	0.39	47.82	---
Fagaceae	*Quercus incana* Bartram Tor banj B.Sul.098.UOP	W	T	Bark	Powder, Poultice	O, top.	Bone fracture, urinary disorders	40	0.238	20	0.5	65	V
Fumariaceae	*Fumaria indica* (Hausskn.) Pugsley Papra B.Sul.066.UOP	W	H	Young shoot	Decoction, juice	O	Blood pressure, vomiting, fever, antispasmodic	41	0.244	21	0.51	68.29	D
Juglandaceae	*Juglans regia* L. Ghoz B.Sul.070.UOP	C	T	Bark	Decoction	O	Wound healing, cleaning teeth	32	0.19	12	0.37	53.12	--
Lamiaceae	*Ajuga bracteosa* Wall. Ex Benth. Bote B.Sul.030.UOP	W	H	Whole plant	Decoction, juice	O	Antipyretic, Blood pressure	20	0.119	8	0.4	30	E
	*Mentha longifolia* L. Welany B.Sul.074.UOP	W	H	Leaves	Powder, juice	O	Diarrhea, vomiting, abdominal pain	46	0.273	38	0.82	95.65	I
	*Mentha piperata* Stokes Podina B.Sul.075.UOP	W	H	Leaves	Powder, juice	O	Abdominal pain, indigestion, diarrhea, nausea	42	0.25	21	0.5	73.80	D
	*Mentha spicata* L. Podina B.Sul.076.UOP	W	H	Leaves	Juice, powder	O	Antipyretic, vomiting, hemorrhoid	40	0.238	20	0.5	65	I
	*Otostegia limbata* (Benth.) Boiss. Spin azghai B.Sul.087.UOP	W	S	Leaves	Direct, powder	O, top.	Teeth ache, wound healing	12	0.071	6	0.5	58.33	I
Meliaceae	*Azadirachta indica* L. Meem B.Sul.038.UOP	W	T	Leaves	Decoction	O	Hepatitis, vermicide	45	0.267	36	0.80	95.55	V
	*Melia azedarach* L. Tora bakyana B.Sul.073.UOP	W	T	Leaves, seeds	Decoction	O	Anti septic, Liver disease, laxative	17	0.101	9	0.52	64.70	R
Menispermaceae	*Tinospora cordifolia* (Willd.) Miers Gelo B.Sul.116.UOP	W	H	Leaves, seeds	Powder	O	Antipyretic, liver disorders, diuretic	38	0.226	19	0.5	68.42	V
Moraceae	*Broussonetia papyrifera* (L.) Vent. Shah toot B.Sul.041.UOP	Inv	T	Leaves	Powder	O	Diarrhea	13	0.077	6	0.46	38.46	---
	*Ficus carica* L. Inza */*B.Sul.064.UOP	W	T	Fruit, latex	Direct	O	Removal of wort, stomach disorders	21	0.125	9	0.42	61.9	V
	*Ficus recemosa* L. Ormal B.Sul.065.UOP	C	T	Latex, fruit	Direct	O, top.	Inflammation due to wasp bites	35	0.208	17	0.48	68.57	---
	*Morus alba* L. Spen toot B.Sul.079.UOP	C	T	Fruit	Direct	O	Constipation, increase digestion	30	0.178	13	0.43	60	---
	*Morus nigra* L. Tor too B.Sul.080.UOP	C	T	Fruit	Direct	O	Coughing, laxative, cooling agent	33	0.196	16	0.48	66.66	---
Myrsinaceae	*Myrsine africana* L. Marlorang B.Sul.081.UOP	W	S	Leaves, fruit	Direct	O	Against worms, abdominal pain	29	0.172	11	0.37	51.72	R
	*Wulfenia amherstiana* L. Nar boty B.Sul.125.UOP	W	H	Leaves	Decoction	O	Hypertension, weakness	18	0.107	9	0.5	61.11	R
Nitrariaceae	*Peganum harmala* L. Spelany B.Sul.090.UOP	W	H	Leaves	Direct	O	Obesity	40	0.238	21	0.52	70	V
Nyctaginaceae	*Boerhavia diffusa* L. nom. Cons. Zakhm boty B.Sul.040.UOP	W	H	Root	Poultice	top.	Skin inflammation, ulcer	45	0.267	32	0.71	88.88	R
Oleaceae	*Olea ferruginea* Wall. Ex Aitch. Khona B.Sul.085.UOP	W	T	Branches	Direct	Toothbrush	Toothache	40	0.238	20	0.5	67.5	R
Oxalidaceae	*Oxalis corniculata* L. Taroky B.Sul.088.UOP	W	H	Leaves	Juice, Poultice	O, top.	Removal of wort, stomach disorders, skin inflammation	47	0.279	40	0.85	97.87	D
Papaveraceae	*Papaver rhoeas* L. Zangaly khaskhash B.Sul.089.UOP	W	H	Seed	Decoction	O	Stomachache, indigestion	37	0.22	19	0.51	67.56	V
Phyllanthaceae	*Andrachne cordifolia* L. Chagzi panra B.Sul.033.UOP	W	S	Leaves	Decoction	O	Diabetes	41	0.244	22	0.53	60.97	R
Platanaceae	*Platanus orientalis* L. Cheenar B.Sul.095.UOP	C	T	Bark	Decoction	top.	Acne, pimple	23	0.136	11	0.47	47.82	---
Poaceae	*Cynodon dactylon* (L.) Pers. Kabal B.Sul.055.UOP	W	H	Whole plant	Poultice	top.	Wound healing	16	0.095	8	0.5	56.25	D
	*Sorghum halepense* (L.) Pers. Dadam B.Sul.111.UOP	W	H	Rhizome	Juice, powder	O, top.	Snake bite, anti-inflammatory	12	0.071	3	0.25	33.33	D
Polygonaceae	*Rumex dentatus* L*./*Shalkhy*/*B.Sul.104.UOP	W	H	Leaves	Decoction, Poultice, vegetable	O, top.	Skin rash, wound healing	30	0.178	15	0.5	56.66	I
	*Rumex hastatus* D. Don Taroky B.Sul.105.UOP	W	H	Leaves	Direct, juice, poultice	O, top.	Skin 1010late10, arthritis, purgative	38	0.226	18	0.47	63.15	V
Portulacaceae	*Portulaca oleracea* L. Warkhary B.Sul.096.UOP	W	H	Young shoot	Vegetable, Roast	O	Constipation	29	0.172	15	0.51	65.51	D
Ranunculaceae	*Aconitum violaceum* Jacquem. Ex Stapf Zahar mora B.Sul.023.UOP	W	H	Roots	Juice, powder	O	Arthritis	44	0.261	30	0.68	86.36	E
Rhamnaceae	*Sageretia theezans* Brongn. Mamana B.Sul.106.UOP	W	S	Leaves	Juice	O	Jaundice	43	0.225	22	0.51	67.44	R
	*Ziziphus nummularia* (Burm.f.) Wight and Arn. Ber B.Sul.128.UOP	W	S	Leaves, fruit	Decoction	O	Ulcer, skin infection	19	0.113	8	0.42	57.89	R
	*Ziziphus oxyphylla* Edgew. Elany B.Sul.129.UOP	W	S	Root, Fruit	Powder, decoction	O	Loss of appetite, constipation, diabetes	37	0.22	18	0.48	64.86	V
Rosaceae	*Duchesnea indica* (Jacks.) Focke Zmaky toot B.Sul.060.UOP	W	H	Fruit	Decoction, direct	O	Sore throat, coughing	16	0.095	7	0.43	50	I
	*Pyrus pashia* L. Tangy B.Sul.097.UOP	C	T	Fruit	Direct	O	Coughing, weakness	30	0.178	14	0.46	56.66	---
	*Rosa damascena* Mill. Palwarai B.Sul.101.UOP	W	S	Petals	Juice	O	Diabetes	37	0.22	19	0.51	54.05	V
	*Rosa webbiana* Wall. Ex Royle Zangaly gulab B.Sul.102.UOP	W	S	inflorescence	Powder, direct	O	Memory stimulant, antispasmodic	23	0.136	10	0.43	60.86	I
	*Rubus fruticosus* G.N. Jones Karwara B.Sul.103.UOP	W	S	Fruit	Direct	O	Cooling agent	32	0.19	12	0.37	46.87	R
Rutaceae	*Zanthoxylum armatum* DC. Dambara B.Sul.127.UOP	W	S	Fruit	Direct, powder	O	Gum pain, cooling agent, abdominal pain	46	0.273	39	0.84	95.65	V
Sapindaceae	*Aesculus indica* (Wall. Ex Cambess.) Hook. Jawaz B.Sul.027.UOP	C	T	Bark, seed	Direct, powder	O	Vermifuge	40	0.238	23	0.57	65	---
	*Dodonaea viscosa* (L.) Jacq. Ghorasky B.Sul.058.UOP	W	S	Leaves	Poultice	top.	Bone fracture, sprain, wound healing,	43	0.255	26	0.60	79.06	D
Scrophulariacea	*Verbascum thapsus* L. Kharghogy B.Sul.119.UOP	W	H	Leaves	Juice	Eardrop	Otitis media	16	0.095	8	0.5	56.25	R
Simaroubaceae	*Ailanthus altissima* (Mill.) Swingle Spena bakanra B.Sul.029.UOP	W	T	Bark	Juice	O	Abdominal pain, skin irritation, pimples	41	0.244	22	0.53	51.21	I
Solanaceae	*Datura innoxia* Mill. Datora B.Sul.056.UOP	W	H	Leaves, fruit	Poultice	top.	Pimple, narcotic	27	0.16	11	0.4	44.44	R
	*Solanum nigrum* L. Kachmachu B.Sul.108.UOP	W	H	Leaves, fruit	Juice, Poultice	O, top.	Gonorrhea, skin diseases	41	0.244	21	0.51	68.29	R
	*Solanum surattense* Burm. Marhaghon B.Sul.109.UOP	W	H	Root, leaves	Poultice, decoction, direct	O, top.	Bone fracture, bronchitis, antipyretic,	43	0.255	25	0.58	76.74	V
	*Withania somnifera* (L.) Dunal Kotelal B.Sul.124.UOP	W	H	Leaves	Powder, vegetable	O	Pneumonia, diuretic	46	0.273	37	0.80	93.47	I
Tamaricaceae	*Tamarix aphylla* (L.) Karst. Ghaz B.Sul.113.UOP	C	T	Root, bark	Decoction	O, top.	Toothache, anti-inflammatory	28	0.166	13	0.46	60.71	---
Taxaceae	*Taxus fuana* Nan Li and R.R. Mill Banrya B.Sul.115.UOP	W	T	Leaves, bark	Powder	O	Diabetes, hepatitis, pneumonia	31	0.184	11	0.35	48.38	V
Urticaceae	Debregeasia saeneb (Forssk.) Hepper Ajlai B.Sul.057.UOP	W	S	Leaves	Powder	top.	Dry skin, fatigue	18	0.107	8	0.44	50	R
Verbenaceae	*Vitex negundo* L. Marwandy B.Sul.123.UOP	W	S	Young shoot	Juice	O	Cramps, rheumatism	27	0.16	13	0.48	59.25	I
Violaceae	*Viola canescens* Wall. Banafsh */*B.Sul.120.UOP	W	H	Leaves, rhizome	Poultice	top.	Arthritis, wound healing	34	0.202	17	0.5	67.64	V
	*Viola odorata* L. Banafsha B.Sul.121.UOP	W	H	Leaves	Decoction	O, top.	Emphysema, itchy throat	31	0.184	16	0.51	64.51	V
	*Viola serpens* L. Boote B.Sul.122.UOP	W	H	Root	Juice	O	Jaundice, wound	37	0.22	18	0.48	62.16	E
Zygophyllaceae	*Tribulus terrestris* L. Markundy B.Sul.117.UOP	W	H	Leaves	Juice	O	Tuberculosis, Sore throat	13	0.077	6	0.46	53.84	R
**Gymnosperm**													
Pinaceae	*Cedrus deodara* (Roxb.) G.Don*/*Ranzro*/*B.Sul.048.UOP	W	T	Oil, gum	Direct	O	Refrigerant, anti septic, antipyretic	20	0.119	10	0.5	70	E
	*Pinus roxburghii* Sarg. Nakhtar B.Sul.092.UOP	C	T	Bark, resin	Direct, decoction	O, top.	Antipyretic, urinary diseases, wound healing	17	0.101	7	0.41	64.70	---
	*Pinus wallichiana* A.B. Jacks. Pewoch B.Sul.093.UOP	W	T	Resin, seeds	Direct	top.	Antipyretic, pimples	13	0.077	5	0.38	53.84	I
**Fungi**													
Agaricaceae	*Agaricus campestris* L. Kharyray B.Sul.028.UOP	W	H	Whole plant	Decoction	O	Stimulant, Nutritive	43	0.255	28	0.65	81.39	V
Morchellaceae	*Morchella deliciosa* Fr. Ghuchy B.Sul.077.UOP	W	H	Whole plant	Decoction	O	Infertility, pain, anti-cholesteric	42	0.25	23	0.54	71.42	E
	*Morchella esculenta* Fr. Ghuchy B.Sul.078.UOP	W	H	Whole plant	Decoction	O	Tonic, infertility	46	0.273	40	0.86	100	V

**Key to abbreviations:** W, wild; C, cultivated; I, invasive; H, herb; S, shrub; T, Tree; ROA, Route of administration; O, oral; top, topical; FC, frequency citation; RFC, relative frequency of citation; UR, use report; UV, use value; FL, fidelity level; CS, Conservation status; D, dominant; E, Endangered; I, infrequent; R, rare; V, vulnerable.

**Table 4:** Informant consensus factor (FCI) by categories of ailments in the study area.

Ailments Category	Nur.	% of Use Reports	Nt.	% of Species	Nur-Nt.	Nur-1	FCI
Gastro-intestinal disorder	137	18.792	75	68.8	62	136	0.45
Respiratory disorders	110	15.089	62	56.88	48	109	0.44
Glandular disorders	104	14.266	58	53.21	46	103	0.44
Cardiovascular disorders	82	11.248	47	43.11	35	81	0.43
Analgesic, Antipyretic and Refrigerant	51	6.995	35	32.11	16	50	0.32
Dermatological disorders	40	5.486	32	29.35	8	39	0.20
Hepatic disorders	33	4.526	27	24.77	6	32	0.18
Body energizers	30	4.115	26	23.85	4	29	0.13
Urologic disorders	27	3.703	22	20.18	5	26	0.19
Nervous disorders	25	3.429	20	18.34	5	24	0.20
Muscles and Skeletal disorders	22	3.017	19	17.43	3	21	0.14
Cancer	21	2.88	21	19.26	0	20	0.00
Ophthalmic disorders	19	2.606	17	15.59	2	18	0.11
Sexual diseases	17	2.331	16	14.67	1	16	0.06
Acoustic disorders	11	1.508	10	9.17	1	10	0.10
**Mean FCI**	**-**	**-**	**-**	**-**	**-**	**-**	**0.226**

Nur, Total use report; Nt, Total number of species used in a disease category; FCI, Informant consensus factor.

**Table 5:** Family wise distribution of medicinal plants and fungi in the study area.

Family	No. of Genera	% of Etycontribution	No. of Species	% of Contribution	FIV
Asteraceae	6	6.38	6	5.50	82.73
Rosaceae	4	4.25	5	4.58	82.14
Lamiaceae	3	3.19	5	4.58	95.23
Moraceae	3	3.19	5	4.58	78.57
Solanaceae	3	3.19	4	3.66	93.45
Fabaceae	3	3.19	3	2.75	64.87
Pinaceae	2	2.12	3	2.75	29.76
Euphorbiaceae	2	2.12	3	2.75	41.07
Rhamnaceae	2	2.12	3	2.75	58.92
Violaceae	1	1.06	3	2.75	60.71
Amaranthaceae	2	2.12	2	1.83	16.07
Amaryllidaceae	2	2.12	2	1.83	33.92
Apocynaceae	2	2.12	2	1.83	34.52
Brassicaceae	2	2.12	2	1.83	35.11
Cannabaceae	2	2.12	2	1.83	42.26
Convolvulaceae	2	2.12	2	1.83	13.69
Ericaceae	2	2.12	2	1.83	33.92
Meliaceae	2	2.12	2	1.83	36.9
Myrsinaceae	2	2.12	2	1.83	27.97
Poaceae	2	2.12	2	1.83	16.66
Sapindaceae	2	2.12	2	1.83	49.4
Asparagaceae	1	1.06	2	1.83	25
Morchellaceae	1	1.06	2	1.83	52.38
Polygonaceae	1	1.06	2	1.83	40.47
Pteridaceae	1	1.06	2	1.83	32.73
Acanthaceae	1	1.06	1	0.91	17.85
Acoraceae	1	1.06	1	0.91	5.95
Agaricaceae	1	1.06	1	0.91	25.59
Anacardiaceae	1	1.06	1	0.91	25.59
Apiaceae	1	1.06	1	0.91	16.66
Araliaceae	1	1.06	1	0.91	20.83
Asclepiadaceae	1	1.06	1	0.91	12.5
Asphodelaceae	1	1.06	1	0.91	22.61
Berberidaceae	1	1.06	1	0.91	26.78
Boraginaceae	1	1.06	1	0.91	22.61
Buxaceae	1	1.06	1	0.91	23.21
Cactaceae	1	1.06	1	0.91	23.8
Caesalpiniaceae	1	1.06	1	0.91	25
Caryophyllaceae	1	1.06	1	0.91	19.04
Celastraceae	1	1.06	1	0.91	16.66
Chenopodiaceae	1	1.06	1	0.91	11.3
Dryopteridaceae	1	1.06	1	0.91	25.59
Equisetaceae	1	1.06	1	0.91	15.47
Fagaceae	1	1.06	1	0.91	23.8
Fumariaceae	1	1.06	1	0.91	24.4
Juglandaceae	1	1.06	1	0.91	19.04
Menispermaceae	1	1.06	1	0.91	22.61
Nitrariaceae	1	1.06	1	0.91	23.80
Nyctaginaceae	1	1.06	1	0.91	26.78
Oleaceae	1	1.06	1	0.91	23.8
Oxalidaceae	1	1.06	1	0.91	27.97
Papaveraceae	1	1.06	1	0.91	22.02
Phyllanthaceae	1	1.06	1	0.91	24.4
Platanaceae	1	1.06	1	0.91	13.69
Portulacaceae	1	1.06	1	0.91	17.26
Ranunculaceae	1	1.06	1	0.91	26.19
Rutaceae	1	1.06	1	0.91	27.38
Scrophulariaceae	1	1.06	1	0.91	9.52
Simaroubaceae	1	1.06	1	0.91	24.4
Tamaricaceae	1	1.06	1	0.91	16.66
Taxaceae	1	1.06	1	0.91	18.45
Urticaceae	1	1.06	1	0.91	10.71
Verbenaceae	1	1.06	1	0.91	16.07
Zygophyllaceae	1	1.06	1	0.91	7.73
**Total**	**94**	**100**	**109**	**100**	**-**
